# Dietary DHA‐Enriched Phosphatidylcholine Enhances Muscle Health and Intestinal Barrier Function by Relieving Apoptosis and Oxidative Stress in Largemouth Bass (*Micropterus salmoides*)

**DOI:** 10.1155/anu/3126231

**Published:** 2026-07-09

**Authors:** Qiang Chen, Tianli Ren, Jinqi Xu, Yunping Tang, Runzhe Zhang, Jiteng Wang, Tao Han

**Affiliations:** ^1^ Department of Aquaculture, Zhejiang Ocean University, Zhoushan, 316022, China, zjou.edu.cn; ^2^ Zhejiang Provincial Engineering Technology Research Center of Marine Biomedical Products, School of Food and Pharmacy, Zhejiang Ocean University, Zhoushan, 316022, China, zjou.edu.cn

**Keywords:** antioxidant capacity, cell apoptosis, DHA-enriched phosphatidylcholine, intestinal flora, largemouth bass

## Abstract

Phosphatidylcholine (PC) is critical for aquatic feed, but the physiological functions of marine‐derived PC remain unclear. This study explored the regulatory role of Atlantic herring (*Clupea harengus*) egg‐derived DHA‐enriched PC (DHA‐PC) in healthy farming of largemouth bass. An 8‐week trial was conducted on juvenile largemouth bass (initial body weight: 4.31 ± 0.038 g) with 0% (control group), 3% and 6% DHA‐PC supplementation. Results showed that DHA‐PC enhanced serum immune indicators (alkline phosphatase (AKP) activity and albumin (ALB) content) and reduced alanine aminotransferase (ALT) activity, while improving antioxidant capacity (increased reduced glutathione (GSH) content, superoxide dismutase (SOD) activity, total antioxidant capacity (T‐AOC) activity and decreased malondialdehyde (MDA) content) in multiple tissues. A total of 496 and 673 differentially expressed genes (DEGs) were identified in muscle transcriptomics between the CON group and the MDHAPC group, and between the CON group and the HDHAPC group, respectively, with enriched apoptosis‐related mitogen‐activated protein kinase/forkhead box O transcription factor (MAPK/FOXO) pathways. Quantitative real‐time polymerase chain reaction (qRT‐PCR) confirmed downregulated apoptosis genes (*nfat2*, *jund*, *ap1*, etc.) by DHA‐PC. DHA‐PC increased the activity of lipase and increased the mRNA expression of *lpl* and *atgl* in the intestine. In addition, DHA‐PC optimized intestinal structure, upregulated tight junction/antioxidant genes (*zo-1*, *claudin-1*, *cat* and *nrf2*), downregulated inflammatory genes (*il-1β* and *tlr2*), and modulated intestinal flora (increased beneficial bacteria and reduced pathogens). In conclusion, dietary DHA‐PC improves muscle and intestinal health via the “intestinal‐muscle axis”, providing a theoretical basis for its application as a novel nutritional strategy in aquaculture. Moreover, comparative analysis revealed that 3% DHA‐PC supplementation was sufficient to achieve significant antioxidant effects, whereas 6% DHA‐PC was more effective in optimizing intestinal microbial community structure.

## 1. Introduction

With the increasing global population and rising living standards, the demand for high‐quality animal protein continues to grow. Hence, healthy farming practices are a crucial development direction in aquaculture that enhances the growth performance and productivity of animals but also ensure their immune health and product quality [1]. However, traditional farming systems face significant challenges, particularly regarding disease control and the overuse of antibiotics, which severely threaten the sustainable development of aquaculture. In recent years, antibiotic misuse in farming has led to increased antibiotic‐resistant bacteria and food safety concerns [2]. To address these challenges, relevant governmental authorities have implemented antibiotic restriction policies to limit and prohibit antibiotic supplementation in animal feed [3]. Within this context, developing nutritional supplements to enhance the immunity and health status of farmed animals has become an urgent industry requirement.

Maintaining healthy aquaculture hinges critically on enhancing the antioxidant capacity of animals as severe oxidative stress can disrupt tissue structure and readily induce metabolic disorders [4]. Oxidative stress can be triggered by environmental conditions, bacteria, and feed components [5–7]. Among feed constituents, lipids represent a potential factor exacerbating oxidative stress [8]. Lipid peroxidation products (such as malondialdehyde (MDA) and 4‐hydroxynonenal (HNE)) damage the fluidity and permeability of cell membranes, which ultimately lead to alterations in the cellular structure and function [9]. Nevertheless, it is undeniable that certain lipid molecules, like docosahexaenoic acid (DHA) and phosphatidylcholine (PC), are themselves potent antioxidants [10, 11]. Primarily, DHA is an essential nutrient for marine fish growth and development, particularly during the larval and juvenile stages [12]. Furthermore, DHA possesses notable antioxidant and anti‐inflammatory properties. Research indicates that DHA can mitigate inflammatory damage by inhibiting the nuclear factor kappa‐B (NF‐*κ*B) signaling pathway [13]. Concurrently, dietary DHA supplementation not only enhances immunomodulatory capacity but also improves intestinal health, reducing the incidence of inflammatory diseases [14].

PC, a vital phospholipid component ubiquitously present in cell membranes, performs diverse physiological functions and promotes fish growth and development [15]. It not only provides choline, facilitating lipid metabolism and liver health but also exhibits antioxidant and anti‐inflammatory effects [16]. Studies showed that PC can modulate lipid metabolism and reduce oxidative stress damage by activating peroxisome proliferator‐activated receptors (PPARs) [17]. Beyond conventional sources like soybeans and eggs, processing by‐products from marine aquatic products serve as significant sources of PC [18]. Marine‐derived PC often contains higher levels of DHA, forming a unique DHA‐enriched PC (DHA‐PC). Utilizing bio‐enzymatic technology, our laboratory has successfully extracted DHA‐PC from Atlantic herring (*Clupea harengus*) roe [19]. The combination of DHA and PC in DHA‐PC confers significant synergistic effects. Integrating the benefits of both DHA and PC, DHA‐PC retains their antioxidant and anti‐inflammatory characteristics. In addition, owing to its unique molecular structure, the bioavailability and targeting efficiency of DHA‐PC are efficient, thereby offering more comprehensive support for the health of farmed animals [20].

The largemouth bass (*Micropterus salmoides*) is extensively cultured worldwide as a significant economic species due to its rapid growth and high nutritional value. However, increasing farming densities and deteriorating aquatic environmental conditions have led to prominent health challenges in this species, which limit the sustainable development of its aquaculture industry [21]. Against this backdrop, this study aims to investigate the potential of DHA‐PC as a nutritional supplement in largemouth bass feed. The results revealed that DHA‐PC exerts systemic health benefits by reducing oxidative stress, inhibiting apoptosis via mitogen‐activated protein kinase/forkhead box O transcription factor (MAPK/FOXO) pathways in muscle, and improving intestinal structure and microbiota, thereby contributing to an integrated intestinal–muscle regulatory axis. This research is expected to facilitate the broader application of DHA‐PC as a novel nutritional additive, offering innovative solutions for healthy aquaculture and food safety.

## 2. Materials and Methods

### 2.1. Ethics Statement

In the present study, all fish experiments were conducted under the supervision and inspection of Animal Experimental Ethical Inspection and Institutional Animals Care and Use Committee of Zhejiang Ocean University.

### 2.2. Feed Production

Three isonitrogenous (crude protein: 48%) and isolipidic (crude lipid: 12%) experimental diets were formulated. Fish meal, soybean meal, and chicken meal were used as the main protein sources, while fish oil, soybean oil, and soybean phospholipid powder served as the main lipid sources. According to the phospholipid requirement of largemouth bass published before [22–24], we supplemented the basal diet with 0%, 3%, or 6% DHA‐PC (wherein DHA‑PC was obtained from ECA Healthcare Inc (Shanghai, China) and prepared according to the method described by Xu et al. [25]); three experimental groups were established and designated as CON, MDHAPC, and HDHAPC, respectively (Table 1). The feed preparation process involved grinding and sieving the raw materials to achieve uniform particle size, followed by stepwise mixing of ingredients in an ascending order of inclusion. Soybean oil and the corresponding proportion of DHA‐PC were then thoroughly blended into the mixture. An appropriate amount of distilled water was added for cold pelleting. The resulting pellet feed was dried at a constant temperature of 45°C for 12 h and stored at –20°C until use.

**Table 1:** Experimental feed formula for largemouth bass (%, dry weight)^**1**^ [26].

Ingredients (%)	CON	MDHAPC	HDHAPC
Fish meal	40	40	40
Soybean meal	25	25	25
Chicken meal	7	7	7
Microcrystalline cellulose	4.05	4.05	4.05
Corn starch	10	10	10
Soybean oil	8	5	2
DHA‐PC^2^	0	3	6
Choline chloride	0.4	0.4	0.4
Vitamin premix^3^	1	1	1
Mineral premix^4^	1	1	1
Monocalcium phosphate	1	1	1
Vitamin C	0.05	0.05	0.05
Sodium alginate	2.5	2.5	2.5
Total	100	100	100
Nutrient composition (dry matter, %)			
Crude protein	48.35	47.97	47.77
Crude lipid	12.10	11.98	12.31

^1^CON, control group; HDHAPC, diet supplementation with 6% DHA‐PC; MDHAPC, diet supplementation with 3% DHA‐PC.

^2^DHA‐PC: DHA‐enriched phosphatidylcholine extracted from herring eggs.

^3^Vitamin premix (g/kg): The components of vitamin premix are as follows, Vitamin A, 2.31; Vitamin D3, 2.02; Vitamin E, 20.00; Vitamin K3, 1.20; Vitamin B5, 10.87; Inositol, 15.00; Niacin, 14.00; Vitamin B6, 3.04; Vitamin B2, 3.00; Vitamin B1, 3.26; Biotin, 0.15; Folic acid, 0.60; Vitamin B12, 0.02; Cellulose, 924.53.

^4^Mineral premix (g/kg): The components of mineral premix are as follows, NaCl, 363.88; MgSO_4_⋅7H_2_O, 586.67; FeSO_4_⋅7H_2_O, 22.22; AlCl_3_⋅6H_2_O, 0.67; KI, 0.67; CuSO_4_⋅5H_2_O, 2.22; MnSO_4_, 4.67; CoCl⋅6H_2_O, 0.86; ZnSO_4_⋅7H_2_O, 18.09; Na_2_SeO_3_, 0.06.

### 2.3. Experimental Fish Rearing and Sample Collection

Prior to the experiment, largemouth bass were acclimated in an indoor recirculating aquaculture system at Zhejiang Ocean University for 2 weeks. During acclimation, fish were fed the CON basal diet. Following acclimation, the fish were fasted for 24 h, after which 180 individuals with uniform body size (initial body weight: 4.31 ± 0.038 g) and good health were selected and randomly distributed into nine 750 L cylindrical tanks (20 fish per tank), each treatment with three replicates. The formal feeding trial lasted for 8 weeks. Fish were fed twice daily (8:00 and 17:00) to apparent satiation. Throughout the trial, water quality was maintained as follows: temperature 24–26°C, dissolved oxygen ≥ 8.0 mg/L, pH 7.7–8.3, ammonia nitrogen < 0.5 mg/L, and nitrite < 0.1 mg/L to ensure stable rearing conditions.

Following the completion of the feeding trial, a 24 h fasting period was conducted for the fish, which were subsequently anesthetized using MS‐222. From each tank, three fish were randomly selected for blood collection via the caudal vein. Blood samples were processed through centrifugation at 4°C and 6000 r/min for 10 min; the resulting serum was then moved to a –80°C freezer to await analysis. An additional six fish per tank were dissected to rapidly collect intestinal, hepatic, and muscle tissues. These samples were immediately snap‐frozen in liquid nitrogen before being placed in ultra‐low temperature storage. For the histological portion of the study, the foregut and muscle were preserved in a 4% paraformaldehyde solution for 24 h to fix the tissue structure.

### 2.4. Determination of Biochemical Indices and Observation of Histological Sections

Serum samples were appropriately diluted before analysis. Specifically, serum samples were diluted 10‐fold for the determination of acid phosphatase (ACP) and alkline phosphatase (AKP) activities, while undiluted serum was used for the analysis of aspartate aminotransferase (AST), alanine aminotransferase (ALT), albumin (ALB), reduced glutathione (GSH), total antioxidant capacity (T‐AOC), and superoxide dismutase (SOD). The corresponding dilution factor was multiplied in the calculation formula to obtain the final enzyme activity concentrations. For the analysis of biochemical indicators in intestinal, muscle, and hepatic tissues, homogenates were first prepared. Precisely 100 mg of tissue was weighed and homogenized with a ninefold volume of physiological saline. The homogenate was then centrifuged at 2500 r/min for 10 min at 4°C, and the resulting supernatant was used for subsequent assays. Assay kits for immune‐related indicators (ACP (cat. number A060‐2‐1), AKP (cat. number A059‐2‐2), ALB (cat. number A028‐2‐1), ALT (cat. number C009‐2‐1), AST (cat. number C010‐2‐1)), antioxidant‐related indicators (T‐AOC (cat. number A015‐2‐1), GSH (cat. number A006‐2‐1), SOD (cat. number A001‐3‐2), MDA (cat. number A003‐1‐2)) and enzyme‐related indicators (LPS (lipase, cat. number A054‐2‐1), AMS (Amylase, cat. number C016‐1‐2), trypsin (cat. number A080‐2‐2)) were purchased from the Nanjing Jiancheng Bioengineering Institute (Jiangsu, China). All procedures were performed in strict accordance with the manufacturers’ instructions. The moisture, crude protein, and crude lipid contents in muscle were determined with a freeze‐dryer (LL1500, Thermo Fisher Scientific, USA), Kjeldahl nitrogen analyzer (K355/K437, BUCHI, Flawil, Switzerland), and Soxhlet extraction (E‐816, BUCHI, Flawil, Switzerland), respectively, according to the standard methods of the Association of Official Analytical Chemists (AOAC) [27].

Fresh intestinal tissue and muscle samples were fixed in 4% paraformaldehyde for a 24‐h period. These tissues were moved through a process of gradient dehydration with ethanol and embedded in paraffin so that 5 μm slices could be sectioned. The resulting sections were stained with hematoxylin and eosin (H&E) and were eventually mounted using neutral balsam. For the final analysis, the stained slides were viewed and photographed with an Olympus BX53 microscope (Japan).

### 2.5. RNA Extraction, Reverse Transcription, and Real‐Time Quantitative PCR

Total RNA from the intestine and muscle was isolated using Trizol reagent (Vazyme Biotech Co., Ltd., Nanjing, China). For this process, about 100–150 mg of tissue was broken down in 1 mL of Trizol and spun at 12,000 × *g* for 10 min. The top liquid was moved to a new tube and mixed with 200 μL of chloroform by shaking it hard for 30 sec; this mixture was spun again at 12,000 × *g* for 15 min. The clear water‐like layer was combined with 400 μL of isopropanol and sat on ice for 10 min before a final 10‐min spin at 12,000 × g. The leftover RNA pellet was cleaned twice with 75% ethanol and was eventually dissolved in nuclease‐free water. To check the quality, the RNA was run on a 1% agarose gel, and the amount was checked with a NanoDrop® ND2000 machine. This RNA was turned into cDNA with a special kit, and the levels of specific genes were measured using a real‐time PCR system with SYBR qPCR Master Mix (Vazyme). The primers for these genes were created from public records and made by Sangon Biotech (Table S1). For the calculations, *β-actin* was chosen as the steady internal control, and the final results for gene levels were found using the 2^−ΔΔCt^ method [28].

### 2.6. Transcriptomic Analysis of Muscle

Muscle tissue samples from the CON, MDHAPC, and HDHAPC groups of largemouth bass (stored at –80 °C) were sent to Guangzhou Gene‐denovo Biotechnology Co., Ltd. (Guang Dong, China) for transcriptome sequencing (RNA‑Seq). The main procedures included total RNA extraction from tissues, concentration measurement, and integrity assessment by agarose gel electrophoresis, followed by reverse transcription of RNA into cDNA and construction of sequencing libraries through fragmentation, end‑repair, and adapter ligation. Library nucleic acid concentration was measured using a Nanodrop 2000, and library quality was evaluated with an Agilent 2100 Bioanalyzer LabChip GX system. Sequencing was performed on an Illumina NovaSeq 6000 platform. To meet the requirements of downstream analysis, raw data were subjected to quality control to remove low‑quality reads. Subsequently, transcriptome alignment and quantification were conducted based on the largemouth bass reference genome. Differentially expressed genes (DEGs) between the two groups were identified using DESeq2, and functional enrichment analysis of the DEGs in Gene Ontology (GO) and Kyoto Encyclopedia of Genes and Genomes (KEGG) pathways was performed with the OmicShare platform.

### 2.7. 16S rRNA Sequencing and Microbiome Analysis

Intestinal tissue samples were stored in 2 mL cryotubes at –80°C. Six samples each from the CON, MDHAPC, and HDHAPC groups were randomly selected for 16S rRNA gene sequencing to analyze the composition and structural characteristics of the gut microbiota in largemouth bass. Sequencing was performed by Guangzhou Gene‐denovo Biotechnology Co., Ltd. Further statistical analysis of the microbial community was conducted in the OmicShare platform, including calculation of *α*‑diversity indices (e.g., abundance‐based coverage estimator (ACE) and Chao1), comparison of species abundance distributions, and significance testing of inter‑group differences.

### 2.8. Data Statistical Analysis

Experimental data are expressed as the mean ± standard error of the mean (S.E.M.). Statistical analysis was performed using SPSS 23.0 software, and differences among groups were compared using Tukey’s test. The *p* value < 0.05 was considered statistically significant.

## 3. Results

### 3.1. Effects of DHA‐PC on Oxidative Stress‐Related Indexes in Various Tissues

**Figure 1:**
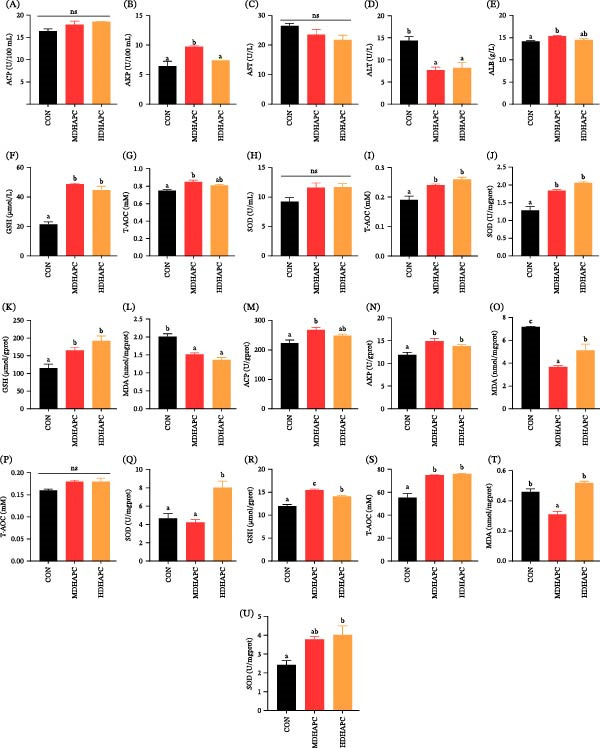
Enzyme activities in the serum (A–H), liver (I–N), intestine (O–Q) and muscle (R–U) (mean ± S. E. M.; *n* = 3). The data were analyzed for significance by Tukey’s test and the different letters marked among the columns indicated that there was significance (*p* < 0.05). ACP, acid phosphatase; AKP, alkline phosphatase; AST, aspartate aminotransferase; ALT, alanine aminotransferase; ALB, albumin; GSH, reduced glutathione; MDA, malonaldehyde; SOD, superoxide dismutase; T‐AOC, total antioxidant capacity. CON: control group; MDHAPC: diet supplementation with 3% DHA‐PC; HDHAPC: diet supplementation with 6% DHA‐PC. DHA‐PC: DHA‐enriched phosphatidylcholine. n.s., not significant.

As shown in Figure 1A–H, in serum, DHA‐PC supplementation did not affect ACP activity; however, the MDHAPC group exhibited significantly elevated AKP activity and ALB content (*p* < 0.05). While DHA‐PC supplementation showed no effect on AST activity, it significantly reduced ALT activity (*p* < 0.05). Regarding antioxidant capacity, DHA‐PC supplementation significantly increased GSH content (*p* < 0.05); the M‐DHAPC group demonstrated significantly enhanced T‐AOC activity (*p* < 0.05), although DHA‐PC supplementation did not significantly affect SOD activity in serum.

As shown in Figure 1I–N, in the liver, both concentrations of DHA‐PC significantly enhanced AKP activity (*p* < 0.05), whereas only the MDHAPC group exhibited significantly elevated ACP activity (*p* < 0.05). In terms of antioxidant capacity, both concentrations of DHA‐PC significantly increased T‐AOC, SOD activity and GSH content (*p* < 0.05), while significantly reducing MDA levels (*p* < 0.05).

As shown in Figure 1O–Q, in the intestine, both concentrations of DHA‐PC significantly reduced MDA content (*p* < 0.05) but did not significantly affect T‐AOC activity; specifically, the HDHAPC group exhibited significantly elevated SOD activity (*p* < 0.05). As shown in Figure 1R–U, in muscle tissue, both concentrations of DHA‐PC significantly increased GSH content and T‐AOC activity (*p* < 0.05); only the MDHAPC group demonstrated a significant reduction in MDA content (*p* < 0.05), while the HDHAPC group showed significantly enhanced SOD activity (*p* < 0.05).

### 3.2. Muscle Transcriptomics Analysis

As shown in Figures 2 and 3, transcriptome analysis of muscle tissue revealed that DHA‐PC supplementation significantly altered gene expression patterns, identifying 496 DEGs (331 upregulated and 165 downregulated in MDHAPC versus CON groups, Figure 2B) and 673 DEGs (249 upregulated and 424 downregulated in HDHAPC versus CON groups, Figure 3B). To elucidate the functional distribution characteristics of these DEGs, functional enrichment analysis was conducted, focusing on five primary categories: metabolism, organismal systems, cellular processes, genetic information processing, and environmental information processing. Within metabolism, lipid, amino acid, and carbohydrate metabolic processes were significantly influenced by DHA‐PC supplementation. Under organismal systems, the endocrine, immune, and circulatory systems exhibited prominent gene enrichment in the MDHAPC and HDHAPC groups. DHA‐PC supplementation significantly impacted transport and catabolism, and cellular growth and death pathways. In genetic information processing, DHA‐PC supplementation induced significant differences in gene enrichment across translation, folding, sorting, and degradation pathways. Within environmental information processing, signal transduction pathways were highly enriched in MDHAPC and HDHAPC groups. To further delineate the biological functions of these DEGs following dietary DHA‐PC supplementation, KEGG pathway enrichment analysis was performed, identifying the 15 significantly enriched pathways. DEGs showed significant enrichment in the MAPK and Foxo signaling pathways under DHA‐PC supplementation. Key factors regulating the Foxo pathway included TGF*β*, Bcl‐6, and KLF2, while TGF*β*, AP1, and JunD were closely associated with the MAPK signaling pathway.

Figure 2Muscle transcriptomics analysis between CON and MDHAPC groups (*n* = 3). (A) Heatmap. (B) Number of differentially expressed genes (DEGs). (C) Volcano plot. (D,E) KEGG pathways. (F) KEGG mapping results of DEGs enriched in FOXO signaling pathway. (G) KEGG mapping results of DEGs enriched in MAPK signaling pathway. In the (F) and (G), green borders are differentially downregulated genes annotated to this pathway; red borders are differentially upregulated genes annotated to this pathway.

**Figure:**
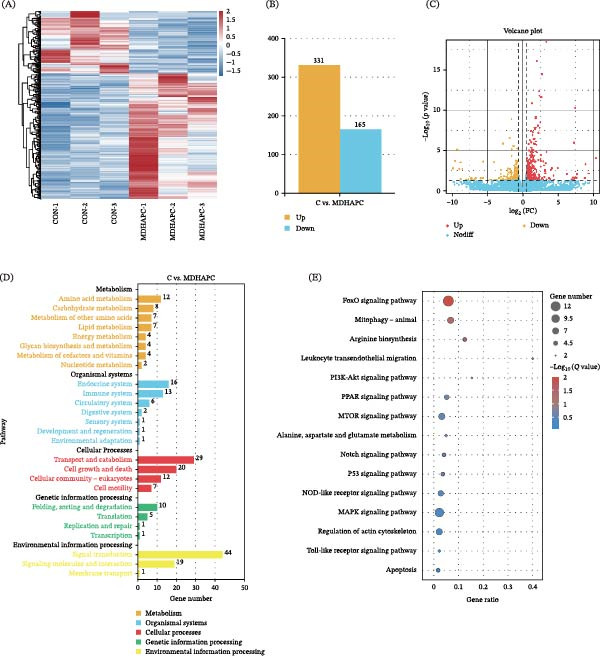

**Figure:**
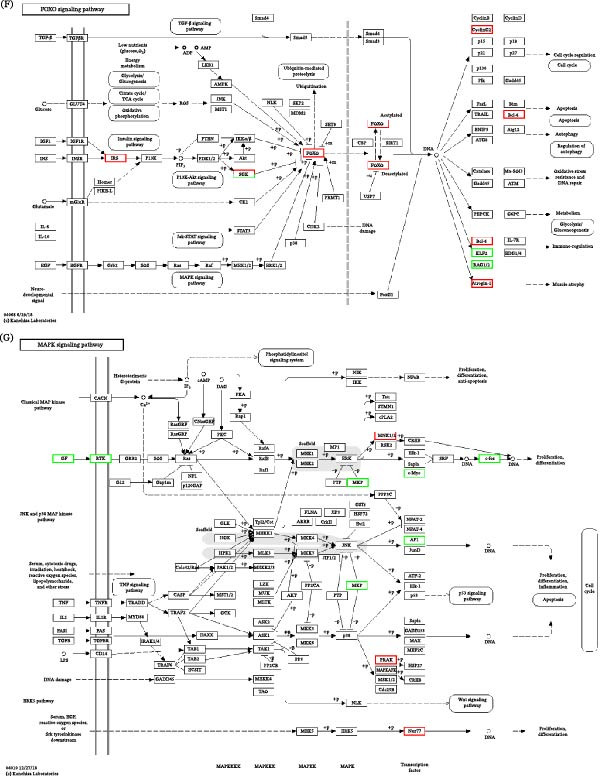

Figure 3Muscle transcriptomics analysis between CON and HDHAPC groups (*n* = 3). (A) Heatmap. (B) Number of differentially expressed genes (DEGs). (C) Volcano plot. (D,E) KEGG pathways. (F) KEGG mapping results of DEGs enriched in FOXO signaling pathway. (G) KEGG mapping results of DEGs enriched in MAPK signaling pathway. In the (F) and (G), green borders are differentially downregulated genes annotated to this pathway; red borders are differentially upregulated genes annotated to this pathway.

**Figure:**
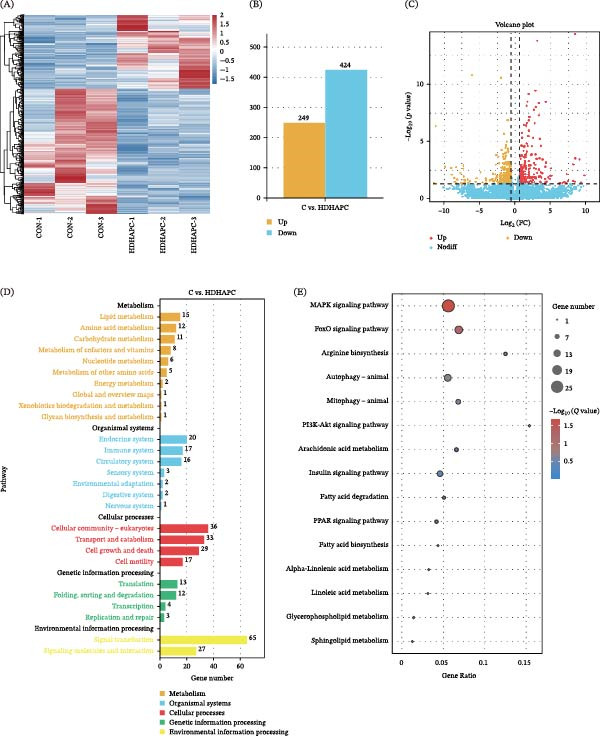

**Figure:**
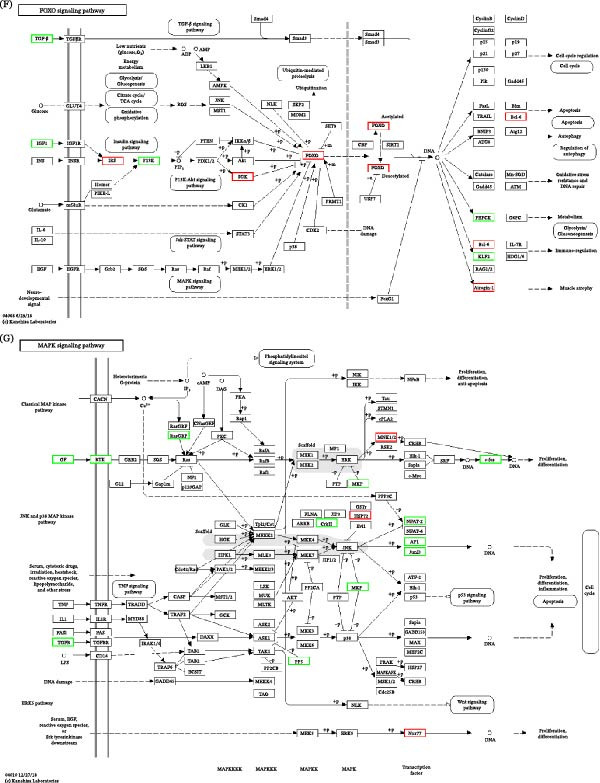

### 3.3. Effects of DHA‐PC on Muscle Nutritional Composition, Apoptosis‐Related Gene Expression, and Histological Structure

**Figure 4:**
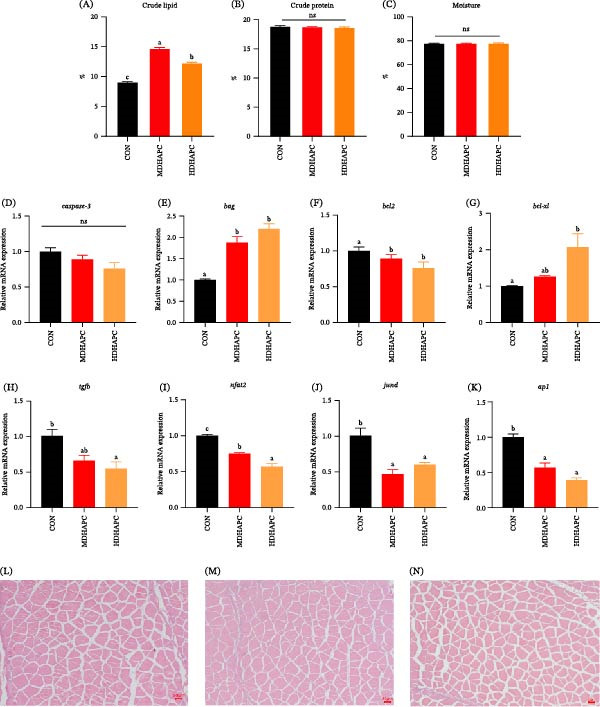
Effects of DHA‐PC supplementation on muscle nutritional composition (A–C), apoptosis‐related gene expression (D–K), and histological structure (L–N, scale bar: 50 μm) (mean ± S. E. M.; *n* = 3). The data were analyzed for significance by Tukey’s test and the different letters marked among the columns indicated that there was significance (*p* < 0.05).

DHA‐PC supplementation significantly increased the crude lipid level in muscle (Figure 4A, *p*  < 0.05). However, DHA‐PC had no significant influence on the crude protein and moisture levels (Figure 4B,C, *p*  > 0.05). Transcriptomic screening identified DEGs significantly enriched in the MAPK and FOXO signaling pathways under DHA‐PC supplementation; KEGG pathway mapping indicated that both pathways converge on the regulation of apoptosis, suggesting that DHA‐PC addition may influence cellular apoptosis. Consequently, the expression of apoptosis‐related genes in muscle tissue was examined. As shown in Figure 4D–K, DHA‐PC supplementation did not significantly affect *caspase3* gene expression but significantly downregulated the mRNA levels of *nfat2*, *bcl2*, *jund*, *ap1*, and *tgfβ* (*p* < 0.05). The expression trends for *nfat2*, *ap1*, *jund*, and *tgfβ* aligned with the transcriptomic analysis, thereby validating the transcriptomic data. Furthermore, DHA‐PC supplementation significantly upregulated *bag* mRNA expression (*p* < 0.05), while the HDHAPC group also exhibited a significant elevation in *bcl-xl* mRNA levels (*p* < 0.05). H&E staining of muscle sections showed that DHA‐PC supplementation increased muscle fiber density (Figure 4L–N).

### 3.4. Effects of DHA‐PC on Intestinal Digestive Enzyme Activities and Lipid Metabolism‐Related Gene Expression in Largemouth Bass

**Figure 5:**
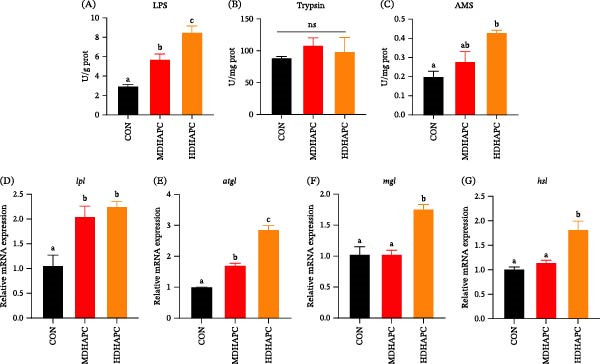
Enzyme activities (A–C) and mRNA expression of genes associated with lipid metabolism in the intestine (D–G) (mean ± S. E. M.; *n* = 3). The data were analyzed for significance by Tukey’s test and the different letters marked among the columns indicated that there was significance (*p* < 0.05). AMS, amylase; LPS, lipase.

As shown in Figure 5, supplementation with DHA‐PC significantly enhanced the activity of LPS (Figure 5A, *p*  < 0.05). However, DHA‐PC supplementation had no significant influence on the activity of trypsin (Figure 5B, *p*  > 0.05). Compared with the CON group, HDHAPC group significantly increased the activity of AMS (Figure 5C, *p*  < 0.05). Both DHA‐PC supplementation groups increased the mRNA expression of *lpl* and *atgl* (Figure 5D,E, *p*  < 0.05). However, only HDHAPC group significantly enhanced the mRNA expression of *mgl* and *hsl* (Figure 5F,G, *p*  < 0.05).

### 3.5. Intestinal Flora Analysis

**Figure 6:**
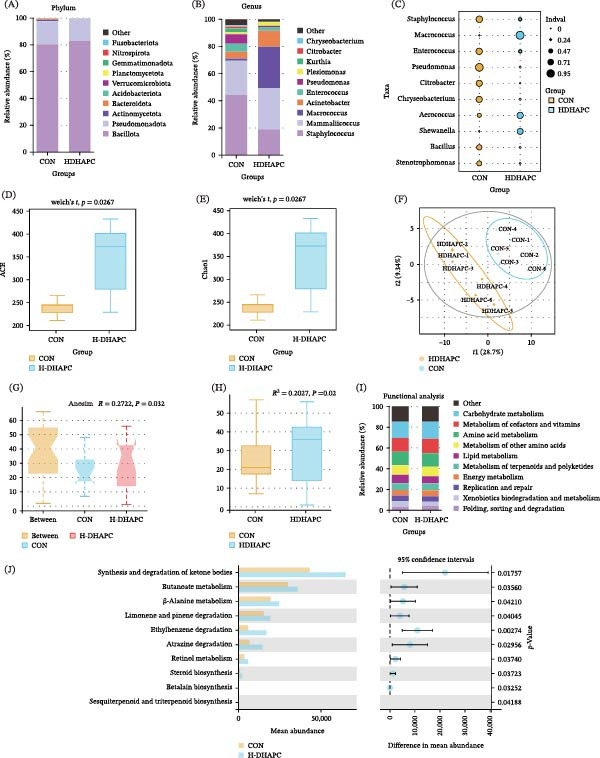
Gut microbiome analysis between CON and HDHAPC groups (*n* = 6). (A, B) The top 10 phyla and genera of intestinal microbiota. (C) Indicator species analysis at genus level. (D, E) *α* diversity analysis. (F) PLS‐DA score plot (*β* diversity statistical testing). (G) Anosim test (*β* diversity statistical testing). (H) Adonis (PERMANOVA) test (*β* diversity statistical testing). (I) Functional analysis of intestinal microbiota. (J) Tax4Fun functional analysis of differential pathways. Welch’s *t*‐test was used to identify the statistical significance with *p*  < 0.05.

Compositional analysis at the phylum level revealed that Pseudomonadota and Bacillota dominated the intestinal microbiota across all groups (Figure S1A and Figure 6A). Indicator species analysis demonstrated that DHA‐PC supplementation effectively altered the microbial composition at the genus level. Both the HDHAPC and MDHAPC groups increased the abundance of *Macrococcus* and *Aerococcus* while decreasing *Staphylococcus* and *Citrobacter* (Figure S1C and Figure 6C). Furthermore, the HDHAPC group specifically increased the abundance of *Shewanella* and decreased *Enterococcus*, *Pseudomonas*, and *Chryseobacterium* (Figure 6C), whereas the MDHAPC group enriched *Mammaliicoccus*, *Enterobacter*, and *Glutamicibacter* and decreased *Paraclostridium*, *Sphingomonas*, and *Stenotrophomonas* (Figure S1C). Alpha diversity analysis indicated that the HDHAPC group significantly elevated both the ACE and Chao1 indices (*p* = 0.0267, Figure 6D,E), suggesting that high‐dose DHA‐PC supplementation significantly enhanced the species diversity of the gut microbiota; a similar increasing trend was also observed in the MDHAPC group (*p* = 0.0634, Figure S1D,E). This distinction in microbial community structure between the treatment groups and the control was further corroborated by PLS‐DA analysis (Figure S1F and Figure 6F). ANOSIM analysis further validated the intergroup differences in gut microbiota structure: the HDHAPC group exhibited significant structural separation from the CON group (ANOSIM *R* = 0.2722, *p* = 0.032, Figure 6G), while the MDHAPC group showed no significant intergroup differentiation (ANOSIM *R* = 0.087, *p* = 0.181, Figure S1G). Additionally, PERMANOVA analysis (*R* ^2^ = 0.2027, *p* = 0.02 for HDHAPC; *R* ^2^ = 0.1526, *p* = 0.091 for MDHAPC) further supported the significant effect of DHA‐PC supplementation on gut microbiota composition, with a stronger effect in the HDHAPC group (Figure S1H and Figure 6H). Functional analysis revealed that the differentially abundant gut microbiota in both groups were primarily enriched in shared pathways related to the synthesis and degradation of ketone bodies, butanoate metabolism, and beta‐alanine metabolism, with the MDHAPC group showing additional enrichment in propanoate metabolism and fatty acid degradation (Figure S1I,J and Figure 6I,J).

### 3.6. Effects of DHA‐PC on the Expression of Genes Related to Intestinal Antioxidant and Inflammatory Response

**Figure 7:**
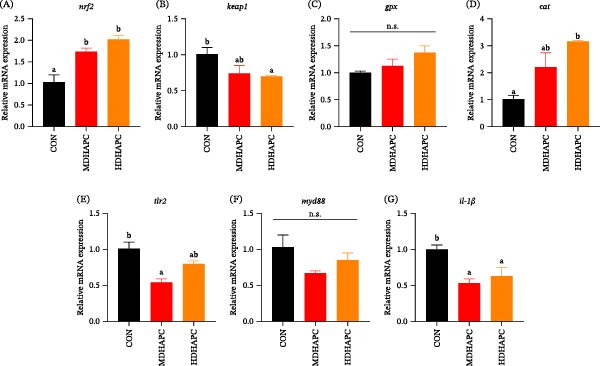
The mRNA expression of genes associated with antioxidant system (A–D) and inflammation (E–G) in the intestine (mean ± S. E. M.; *n* = 3). The data were analyzed for significance by Tukey’s test and the different letters marked among the columns indicated that there was significance (*p* < 0.05).

As shown in Figure 7, Within the intestine, both concentrations of DHA‐PC significantly upregulated *nrf2* mRNA expression levels (*p* < 0.05) but had no significant effect on *gpx* mRNA expression. HDHAPC group significantly suppressed *keap1* mRNA expression while significantly upregulating *cat* mRNA expression (*p* < 0.05). Regarding the inflammatory response, the MDHAPC group significantly downregulated *tlr2* mRNA expression (*p* < 0.05), both concentrations of DHA‐PC significantly suppressed *il-1β* mRNA expression (*p* < 0.05), yet neither concentration significantly affected *myd88* mRNA expression.

### 3.7. Effects of DHA‐PC on the Expression of Gut Barrier‐Related and Apoptosis‐Related Genes and Histological Observation of Intestine

**Figure 8:**
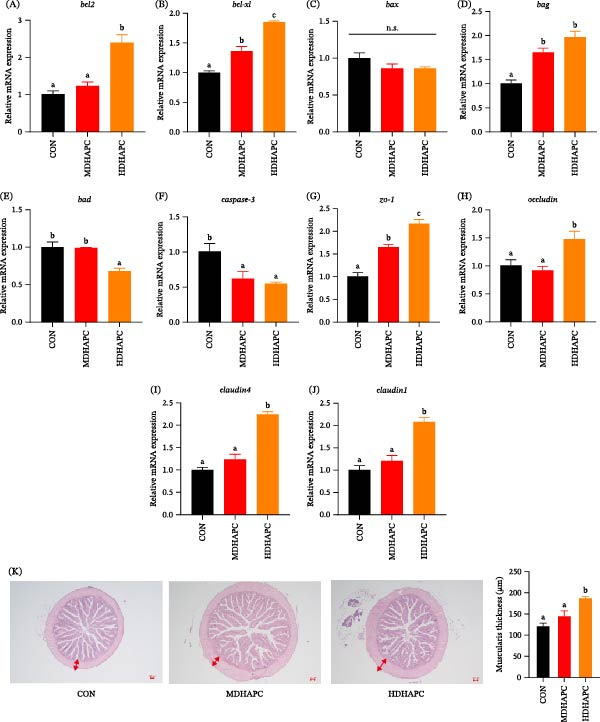
Indicators of intestinal health. (A–F) The mRNA expression of genes associated with apoptosis in the intestine (*n* = 3). (G–J) The mRNA expression of genes associated with tight junction proteins (*n* = 3). (K) Intestinal H&E sections. Scale bar: 100 μm. The red two‐way arrow represents the thickness of the intestinal wall muscle layer. The data were analyzed for significance by Tukey’s test and the different letters marked among the columns indicated that there was significance (*p* < 0.05).

As shown in Figure 8, regarding apoptosis, DHA‐PC supplementation did not significantly affect *bax* mRNA expression. However, both doses of DHA‐PC significantly upregulated *bcl-xl* and *bag* mRNA levels while significantly downregulating *caspase3* mRNA expression (*p* < 0.05). Additionally, HDHAPC group significantly elevated *bcl2* mRNA expression but significantly suppressed *bad* mRNA expression (*p* < 0.05). Concerning intestinal barrier function, both doses of DHA‐PC significantly upregulated *zo-1* mRNA expression, and the HDHAPC group further significantly enhanced the mRNA expression of *claudin1*, *claudin4*, and *occludin* (*p* < 0.05). Intestinal H&E‐stained sections showed DHA‐PC elevated intestinal wall thickness (Figure 8K).

## 4. Discussion

Recognizing the pivotal role of DHA‐PC (extracted from Atlantic herring roe) in sustainable and healthy aquaculture, this study comprehensively evaluated the systemic health benefits of dietary DHA‐PC in largemouth bass. Specifically, we elucidated how DHA‐PC establishes an integrated intestinal–muscle regulatory axis by optimizing gut morphometry and microecology, mitigating oxidative stress, and suppressing muscle apoptosis via the MAPK/FOXO signaling pathways. DHA and PC play crucial roles in enhancing the nonspecific immune function of fish [15, 29]. The nonspecific immune system in fish serves as a vital barrier against invasive pathogens such as viruses and bacteria, and its responsiveness is directly linked to disease resistance and overall health [30]. ACP and AKP are important hydrolases in macrophage lysosomes and involved in the entire process of pathogen recognition, phagocytosis, and clearance [31]. ALB levels reflect the metabolic status and amino acid absorption efficiency of the organism. An increase in ALB level indicates enhanced nonspecific immune function in fish [32]. ALT and AST are sensitive indicators of liver injury in aquatic animals, both of which are released into the bloodstream in large quantities upon hepatocyte damage [33]. In this study, the group supplementation with DHA‐PC showed significantly reduced serum ALT and AST activities, along with significantly increased serum ALB content and hepatic ACP and AKP activities. Previous results from our laboratory have confirmed that DHA‐PC not only effectively alleviates high‐fat‐diet‐induced elevations in serum ALT and AST levels in mice [19] but also significantly enhances hepatic AKP and ACP activities as well as serum ALB content in *Litopenaeus vannamei* [25]. In summary, these cross‐species results demonstrate that dietary DHA‐PC can effectively enhance nonspecific immune function and provide significant protection for liver health in animals.

Under intensive aquaculture, fish frequently suffer from oxidative stress, which seriously threatens the sustainable development of aquaculture. Enhancing the antioxidant capacity of fish through nutritional regulation has become a key strategy for healthy farming. Studies have shown that dietary phospholipids can improve an animal’s resistance to environmental stress and activate the antioxidant defense system to protect organs from oxidative damage [34]. Unlike terrestrial phospholipid sources, marine‐derived PC is typically rich in DHA, forming structurally unique DHA‐PC. This combines the antioxidant effects of DHA to collectively reduce reactive oxygen species (ROS) production and cellular damage, thereby enhancing antioxidant capacity and improving overall antioxidant function [35, 36]. In this study, comprehensive analysis of enzyme activity data from serum, liver, muscle, and intestine revealed that dietary supplementation with DHA‐PC significantly enhanced the antioxidant capacity of largemouth bass. This effect has been consistently validated in related studies on mammals [19] and aquatic animals [25]. Notably, the intestine is a key organ for maintaining systemic redox homeostasis and immune barrier function. The expression levels of antioxidant genes in the intestine can most directly reflect the transcriptional regulation of nutrients on the body’s antioxidant system. Nrf2 is a critical signaling factor that regulates the transcription of antioxidant enzymes in the body [37]. Keap1 is a key sensor in cellular oxidative stress. When ROS levels increase, the expression of the Keap1 gene is downregulated, promoting the translocation of Nrf2 from the cytoplasm to the nucleus, where it binds to the antioxidant response element (ARE) and regulates the expression of downstream antioxidant enzyme genes such as *cat* and *gpx* [38]. The results of this study demonstrate that dietary DHA‐PC significantly increased the expression of *nrf2*, *gpx*, and *cat* genes and downregulated the *keap1* gene expression. This indicates that DHA‐PC may enhance the antioxidant capacity of largemouth bass by activating the Keap1/Nrf2‐ARE signaling pathway.

Fish muscle is an important source of high‐quality protein. Muscle growth represents a complex dynamic process, which is regulated by genetic, environmental, and nutritional factors [39]. For consumers, appropriate lipid deposition in fish muscle can enhance muscle flavor, and the supplementation of DHA‐PC promoted the deposition of crude lipid in muscle. Our previous studies have also confirmed that DHA‐PC supplementation regulated lipid metabolism and reshaped phospholipid metabolism [26], which may partially explain the increase in crude lipid content. On this basis, we conducted research from the perspective of muscle health. In the present study, RNA‐seq analysis revealed that DHA‐PC supplementation significantly enriched the MAPK and FOXO signaling pathways. These pathways play crucial roles in mediating cellular processes such as inflammation, stress response, and apoptosis [40–42]. Further analysis of downstream target genes enriched in the MAPK and FOXO pathways revealed that the apoptotic process was significantly influenced by DHA‐PC. The functions of several key genes are outlined as follows: JUND is a component of the AP‐1 dimer, and NFAT cooperates with AP‐1 to regulate apoptosis [43]. BCL‐6, a transcriptional repressor that binds to the promoter region of P53, inhibits apoptosis by downregulating P53 expression [44]. BCL2 and BCL‐XL belong to the antiapoptotic BCL‐2 family and inhibit apoptosis by preventing mitochondrial membrane permeabilization (MMP) [45]. In this study, DHA‐PC inhibited apoptosis by downregulating the expression of apoptosis‐related genes *nfat2*, *jund*, and *ap-1* and upregulating the expression of the antiapoptotic gene *bcl-6*. Real‐time fluorescence quantitative PCR verified the authenticity of RNA‐seq data. Therefore, DHA‐PC can modulate muscle cell apoptosis in largemouth bass through the MAPK/FOXO cascade. Improvements in the muscle tissue observed by H&E‐stained sections in the present study may also be based on transplantation of apoptotic pathways.

The intestine is not only a central organ for nutrient absorption but also a critical barrier for maintaining immune homeostasis and structural integrity in the body. In the present study, DHA‐PC significantly promoted intestinal digestive ability, which is also based on the enhancement of intestinal structure. Intestinal villus height and intestinal wall thickness are key morphological indicators for evaluating nutrient digestion and absorption in fish [46]. The intestinal physical barrier consists of intestinal epithelial cells and tight‐junction proteins, including transmembrane proteins such as claudins and occludin, as well as the zonula occludens family of peripheral membrane proteins, which are essential for maintaining normal intestinal morphology, physiology, and function [47, 48]. Studies have shown that tight‐junction proteins play a crucial role in the physical barrier function of the fish intestine by dynamically regulating epithelial permeability [49, 50]. Our study demonstrated that DHA‐PC significantly increased the expression levels of genes associated with intestinal physical barrier, thereby reducing intestinal permeability and enhancing intestinal health in fish. The intestinal immune barrier serves as a key frontline defense against pathogen invasion via the gut. In this process, cytokines are core indicators for assessing host immune status and play a vital role in regulating inflammatory responses and immune function [51]. IL‐1*β*, a key proinflammatory cytokine, is regulated by the TLR2/MYD88 signaling pathway [52]. In this study, dietary DHA‐PC downregulated the expression of *tlr2* and *il-1β* genes. These findings are consistent with reports in large yellow croaker [53] and snapper [54], indicating that DHA‐PC may exert immunomodulatory effects by regulating the TLR2‐mediated signaling pathway. In addition, results showed that DHA‐PC may inhibit the activation of apoptotic signaling pathways by modulating the balance of Bcl‐2 family proteins, thereby reducing programmed cell death in the intestinal cells of largemouth bass. Integrating muscle transcriptomics and intestinal functional analyses, the present study indicates that DHA‐PC can coordinate apoptosis‐related pathways across different tissues to maintain systemic homeostasis. The functional synergy established between intestinal and muscle tissues under DHA‐PC regulation helps enhance structural integrity, support normal cell renewal and repair, and ultimately improve the overall health status and growth performance of fish.

The gut microbiota constitutes a complex symbiotic ecosystem capable of modulating host physiology, nutrition, and immune function [55]. Compared to mammals, the intestinal microbial community in fish exhibits higher plasticity, with its composition and metabolic functions being regulatable through nutritional components [56]. Studies in mammals have shown that DHA‑PC can alleviate high‑fat‑diet‑induced non‑alcoholic fatty liver disease and inflammation, as well as promote insulin signaling transduction, by modulating the gut microbiota [19, 57]. In the present study, at the phylum level, the intestinal microbiota of largemouth bass was dominated by *Bacillota* and *Pseudomonadota*, which aligns with the predominant microbial composition reported in other fish species such as red mullet [58] and African catfish [59]. Alpha‑diversity analysis revealed that the ACE and Chao1 indices were significantly higher in the H‑DHAPC group compared to the control. At the genus level, H‐DHAPC supplementation significantly increased the abundance of *Acinetobacter*, *Micrococcus*, and *Shewanella*. These genera have been reported to be closely associated with host immunomodulation and can enhance immune defense in aquatic animals [60–62]. In contrast, *Staphylococcus* is a pathogenic bacterium in fish that can disrupt the immune barrier and induce inflammation during infection [63]. *Enterococcus*, a Gram‑positive, facultatively anaerobic bacterium resistant to harsh environmental conditions, is an infectious agent in fish associated with high mortality rates [64]. In this study, H‐DHAPC supplementation significantly reduced the abundance of Staphylococcus and Enterococcus. These findings indicate that DHA‑PC can effectively maintain intestinal homeostasis and health by modulating the structure of the gut microbial community. Furthermore, it may, through the microbiota‑gut‑muscle axis, shape a more stable and diverse gut microecology, thereby indirectly improving nutrient utilization efficiency, immune responsiveness, and overall disease resistance in fish, ultimately promoting the healthy development of muscle tissue. However, the specific targets and molecular mechanisms within this axis warrant further elucidation through integrated multi‑omics analyses.

In summary, dietary supplementation with DHA‐PC significantly enhanced immune function and antioxidant capacity in largemouth bass, while also inhibiting muscle cell apoptosis and promoting muscle health. Simultaneously, DHA‐PC coordinately regulated the expression of genes related to apoptosis, antioxidation, and inflammation in the intestine, optimized the gut microbiota composition, and improved intestinal structure. These effects collectively constitute a “gut–muscle axis” coregulatory mechanism, achieving systemic integration of multiorgan health benefits. This study provides a theoretical foundation for the application of DHA‐PC as a functional feed additive in sustainable and healthy aquaculture. Future research should focus on reducing production costs of extracting DHA‐PC from fish roe and promoting its large‐scale application in aquafeeds. Developing lipases to achieve the directed catalytic conversion of plant‐derived phospholipid sources with fish oil represents a more economical strategy for the synthesis of DHA‐PC. In that case, graded levels with more details would better determine the optimal inclusion rate of DHA‐PC in the aquaculture diet.

## Author Contributions

**Qiang Chen**: conceptualization, funding acquisition, writing – original draft. **Tianli Ren**: methodology, investigation, software, writing – review and editing. **Jinqi Xu**: data curation, formal analysis, writing – review and editing. **Yunping Tang**: resources, writing – review and editing. **Runzhe Zhang**: methodology, investigation, software. **Jiteng Wang**: project administration, writing – review and editing. **Tao Han**: conceptualization, project administration, writing – review and editing.

## Funding

This work was supported by the Zhejiang Provincial Natural Science Foundation of China (Grant LQ24C190006) and National Natural Science Foundation of China (Grant 32303023).

## Conflicts of Interest

The authors declare no conflicts of interest.

## Supporting Information

Additional supporting information can be found online in the Supporting Information section.

## Supporting information


**Supporting Information** Table S1: lists the primer sequences utilized for quantitative real‐time PCR detection. Figure S1: illustrates the intestinal microbiome comparative analysis of the CON and MDHAPC groups.

## Data Availability

The data that support the findings of this study are available from the corresponding author upon reasonable request.
