# Supplementary material for: Dietary DHA‐Enriched Phosphatidylcholine Enhances Muscle Health and Intestinal Barrier Function by Relieving Apoptosis and Oxidative Stress in Largemouth Bass (Micropterus salmoides)
Source: Aquac Nutr. 2026 Jul 9;2026:3126231. doi: 10.1155/anu/3126231 (PMC13347174; doi:10.1155/anu/3126231)
Supplement: Supplementary file 1 — Supporting Information Table S1: lists the primer sequences utilized for quantitative real‐time PCR detection. Figure S1: illustrates the intestinal microbiome comparative analysis of the CON and MDHAPC groups. [file ANU-2026-3126231-s001.docx]

**Supplementary Table 1 Primer sequences for real-time fluorescent quantitative PCR**

| Genes | forward primers (5’→3’) | reverse primers (5’→3’) | References |
| --- | --- | --- | --- |
| *β-actin* | ATCGCCGCACTGGTTGTTGAC | CCTGTTGGCTTTGGGGTTC | Chen et al., 2012 |
| *gapdh* | ACTGTCACTCCTCCATCTT | CACGGTTGCTGTATCCAA | Chen et al., 2023 |
| *caspase-3* | GCTTCATTCGTCTGTGTTC | CGAAAAAGTGATGTGAGGTA | Liu et al., 2022 |
| *bag* | ATGACCCGAGACACGACAC | CATAACCTGGGCGAAGAAT | Liu et al., 2022 |
| *bcl2* | CCATCCACGACGAACCTG | GGCGTATCGCTGCTCAAACT | Liu et al., 2022 |
| *bcl-xl* | CATCCTCCTTGGCTCTGG | GGGTCTGTTTGCCTTTGG | Liu et al., 2022 |
| *tgfβ* | GCTCAAAGAGAGCGAGGATG | TCCTCTACCATTCGCAATCC | Zhou et al., 2021 |
| *nfat2* | CGCACAAGCGTAACAGATGAC | AGGTGAGTGGTTGGGCGAGTA | XM_038721873.1 |
| *jund* | TTTACGCTACGGTTTATTTGTC | CCAGGCTCTTCATCATTATTTC | XM_038718267.1 |
| *ap1* | CCCGACTATGTCCTTCCCTG | CGGTTCCTCATCCGCTTTCT | XM_038716392.1 |
| *nrf2* | CACCAAAGACAAGCGTAAG | GAAATCATCAACAGGCAGA | Chen et al., 2023 |
| *keap1* | AGACGGCAGGAGATGTTGT | CATGGCTCTGAAGTAGGGG | Chen et al., 2023 |
| *gpx* | CTCCTCAACCAGGCAAAC | ATACCCCCCTCACAACAA | Chen et al., 2023 |
| *cat* | TGAATGGCTATGGCTCTC | AATCTGGGTTGGTGGAAG | Chen et al., 2023 |
| *tlr2* | TCGCTGTTCACCAATCTG | TAGTTCTCCTCTCCATCTGT | Zhang et al., 2022 |
| *myd88* | CTCAACCCCAAGAACACA | CGAAGATCCTCCACAATG | Zhang et al., 2022 |
| *il-1β* | TTGCCATAGAGAGGTTTA | ACACTATATGCTCTTCCA | Zhang et al., 2022 |
| *lpl* | TTCCTCGACCCTCTGAAAGA | GGAGTCAAGTTTGCCAGGAA | Yu et al., 2019 |
| *atgl* | GCTCCCCTACACTCTCCCTCT | CTGCTCTCGAATCCACTCAAC | Yuan et al., 2022 |
| *mgl* | AAGGTTTTTCTGGCGAAGGT | CGTGGAAGTTCAGCTCATCA | Yu et al., 2019 |
| *hsl* | ATCAGAGCTGGAGCACCCTA | GCAGAGGAGAGCAGAAAGGA | Yu et al., 2019 |
| *zo-1* | ATCTCAGCAGGGATTCGACG | CTTTTGCGGTGGCGTTGG | Zhou et al., 2021 |
| *occludin* | CGGCCATTTTACACGCATTGA | CCACACTGTCAGGCTCTTGT | Liu et al., 2022 |
| *claudin4* | TAATCGCTATGGTGGGAGCC | GCCCCGATCTCCATCTTCTG | Zhou et al., 2021 |
| *claudin1* | CCAGGGAAGGGGAGCAATG | GCTCTTTGAACCAGTGCGAC | Zhou et al., 2021 |

**
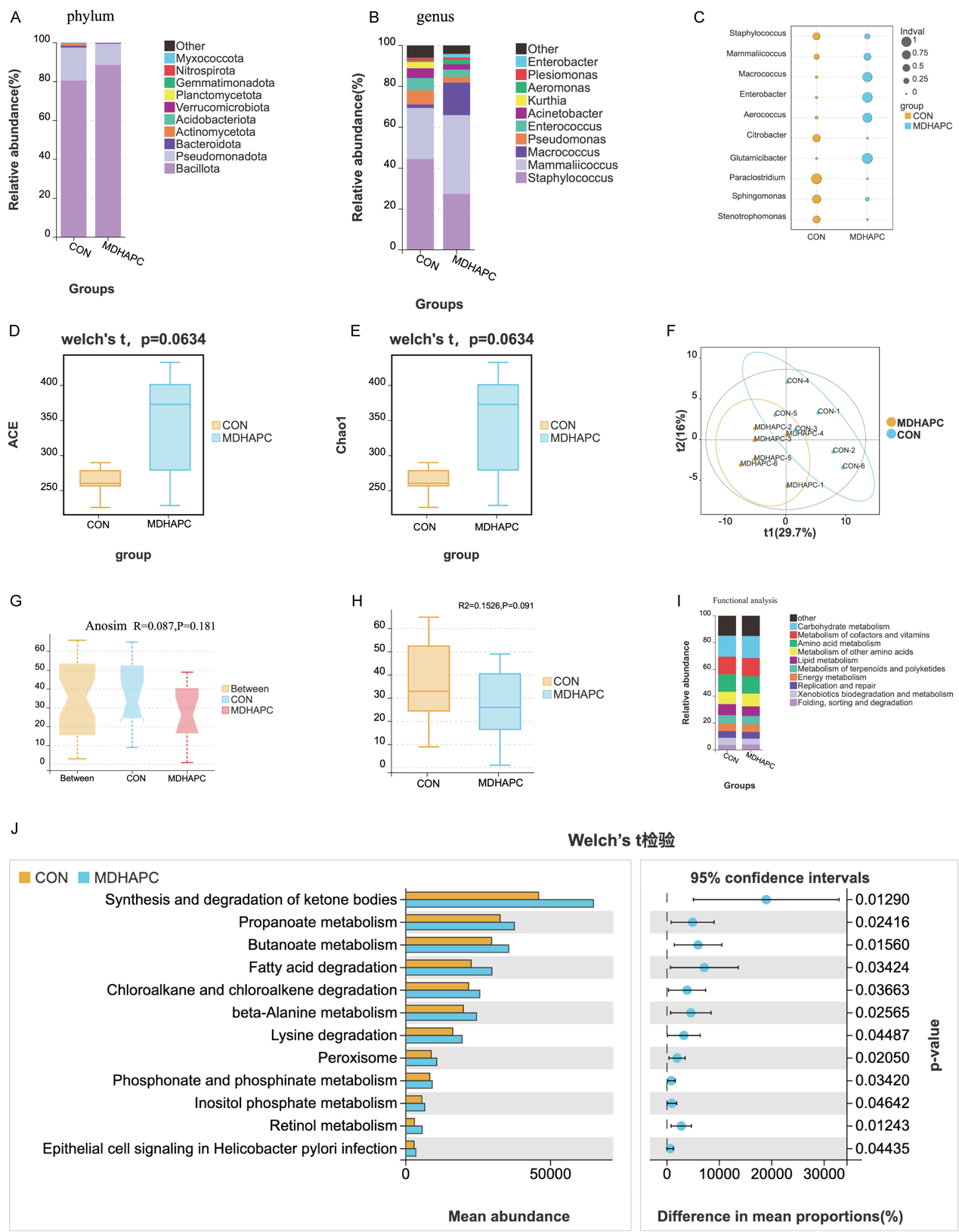
**

**Supplementary Figure 1 Gut microbiome analysis between CON and MDHAPC groups (n = 6).** (A, B) The top 10 phyla and genera of intestinal microbiota. (C) Indicator species analysis at genus level. (D, E) α diversity analysis. (F) PLS-DA score plot (β diversity statistical testing). (G) Anosim test (β diversity statistical testing). (H) Adonis (PERMANOVA) test (β diversity statistical testing) (I) Functional analysis of intestinal microbiota. (J) Tax4Fun functional analysis of differential pathways. Welch’s t-test was used to identify the statistical significance with *P* < 0.05.

**References**

Chen, N.Jin, L., et al. Effects of dietary arginine levels and carbohydrate-to-lipid ratios on mRNA expression of growth-related hormones in largemouth bass, *Micropterus salmoides*. General and Comparative Endocrinology 2012. 179(1), 121-127. https://doi.org/10.1016/j.ygcen.2012.08.004.

Chen, Q.Wang, C., et al. Excessive Substitution of Fish Meal with Fermented Soybean Meal Induces Oxidative Stress by Impairing Glutathione Metabolism in Largemouth Bass (*Micropterus salmoides*). Antioxidants 2023. 12(12), 2096. https://doi.org/10.3390/antiox12122096.

Liu, Y.Huang, H., et al. Effects of dietary non-starch polysaccharides level on the growth, intestinal flora and intestinal health of juvenile largemouth bass *Micropterus salmoides*. Aquaculture 2022. 557, 738343. https://doi.org/10.1016/j.aquaculture.2022.738343.

Yu, H.Liang, X., et al. Dietary supplementation of Grobiotic®-A increases short-term inflammatory responses and improves long-term growth performance and liver health in largemouth bass (*Micropterus salmoides*). Aquaculture 2019. 500, 327-337. https://doi.org/10.1016/j.aquaculture.2018.10.033.

Yuan, Y.Jiang, X., et al. Toxicological impacts of excessive lithium on largemouth bass (*Micropterus salmoides*): Body weight, hepatic lipid accumulation, antioxidant defense and inflammation response. Science of the Total Environment 2022. 841. https://doi.org/10.1016/j.scitotenv.2022.156784.

Zhang, Q.Liang, H., et al. Effects of replacing fishmeal with methanotroph (*Methylococcus capsulatus*, Bath) bacteria meal (FeedKind®) on growth and intestinal health status of juvenile largemouth bass (*Micropterus salmoides*). Fish&Shellfish Immunology 2022. 122, 298-305. https://doi.org/10.1016/j.fsi.2022.02.008.

Zhou, Y.He, G., et al. High dietary starch impairs intestinal health and microbiota of largemouth bass, *Micropterus salmoides*. Aquaculture 2021. 534, 736261. https://doi.org/10.1016/j.aquaculture.2020.736261.
